# Collapsing Glomerulopathy in Brazil: A Nationwide Descriptive Study

**DOI:** 10.1111/nep.70158

**Published:** 2025-12-15

**Authors:** Marcos Adriano Garcia Campos, Precil Diego Miranda de Meneses Neves, Érico Murilo Monteiro Cutrim, Andressa Monteiro Sodré, João Victor Carvalho, Davi Campos Wanderley, Stanley de Almeida Araújo, Ricardo Miranda Borges, Luiz Fernando Onuchic, Andréia Watanabe, Irene de Lourdes Noronha, Vanessa dos Santos Silva, Igor Denizarde Bacelar Marques, Felipe Leite Guedes, José Bruno de Almeida, Rafael Fernandes Vanderlei Vasco, Antônio Monteiro, Francisco Rasiah Ladchumananandasivam, Orlando Vieira Gomes, Antonio Augusto Lima Teixeira Júnior, Karla Cristina Petruccelli Israel, Denise Maria do Nascimento Costa, Fernanda Pinheiro Martin Tapioca, Epitácio Rafael da Luz Neto, Joyce Santos Lages, Natalino Salgado Filho, Gyl Eanes Barros Silva

**Affiliations:** ^1^ Duke Global Health Institute, Duke University Durham North Carolina USA; ^2^ Nephrology Division Medical School, University of São Paulo (FM‐USP) São Paulo Brazil; ^3^ Medical School (HCFMB), São Paulo State University (UNESP) Botucatu Brazil; ^4^ Laboratory Immunofluorescence and Electronic Microscopy University Hospital, Federal University of Maranhão (HU‐UFMA) São Luís Brazil; ^5^ Nephropathology Institute, Federal University of Minas Gerais (UFMG) Belo Horizonte Brazil; ^6^ Nephrology Service, University Hospital, Federal University of Piauí (HU‐UFPI) Teresina Brazil; ^7^ Nephrology Service, Onofre Lopes University Hospital, Federal University of Rio Grande Do Norte (HUOL‐UFRN) Natal Brazil; ^8^ Division of Nephrology University Hospital, Federal University of Alagoas (UFAL) Maceio Brazil; ^9^ Division of Nephrology Maria Aparecida Pedrossian University Hospital, Federal University of Mato Grosso Do Sul (HUMAP–UFMS) Campo Grande Brazil; ^10^ Nephrology Service, Lauro Wanderley University Hospital, Federal University of Paraíba (HULW‐UFPB) João Pessoa Brazil; ^11^ Division of Nephrology University Hospital, Federal University of Vale Do São Francisco (HU‐UNIVASF) Petrolina Brazil; ^12^ Ribeirão Preto Medical School, University of São Paulo (PGGEN‐FMRP‐USP) Ribeirão Preto Brazil; ^13^ Department of Clinical Medicine Federal University of Amazonas (UFAM) Manaus Brazil; ^14^ Nephrology Service, Clinical Hospital, Federal University of Pernambuco (UFPE) Recife Brazil; ^15^ Nephrology Service, Ana Nery Hospital Salvador Brazil

**Keywords:** chronic kidney disease, collapsing glomerulopathy, epidemiology, kidney biopsy, renal replacement therapy

## Abstract

**Aim:**

Collapsing glomerulopathy (CG) is a glomerular disease that progresses rapidly to renal replacement therapy (RRT). Brazil is a conducive site for studies on CG because of its significant burden of infectious diseases and a high frequency of APOL1 risk variants. We described the clinical characteristics, histopathological findings, and outcomes of CG patients.

**Methods:**

This retrospective survey analysed data from 2014 to 2022 from 18 centres of nephrology in all Brazilian regions. CG was diagnosed by kidney biopsy (KB), and the data were collected from medical records: initial symptoms, treatment, evolution to RRT.

**Results:**

A total of 330 patients were included. Most patients were men (61.2%) and of Brown/Black ethnicity (72.7%). The mean age was 32 years. The most common symptoms were loss of renal function (44.2%), nephrotic proteinuria (80.07%), and hematuria (52.31%). KB revealed mild tubular atrophy and interstitial fibrosis (53.3%), moderate‐to‐severe interstitial inflammation (35.9%), and acute tubular necrosis (19.0%). On immunofluorescence, IgM (75.5%) and C3 (81.2%) were found to be most frequently deposited. Secondary etiologies were identified in 94 patients: HIV (13.8%), autoimmune disease (15.9%), and APOL1 high‐risk genotype (48.6%, 35/72). The mean follow‐up period was 13 months. Nephrotic proteinuria persisted in 78.8% of patients, and 30.5% progressed to RRT within 3 months after diagnosis.

**Conclusion:**

CG affects young patients, leading to RRT in one‐third of cases in approximately 3 months. This multicentre study sets the baselines for risk factors and outcomes in the largest population studies on CG.

## Summary at a Glance

- In a study of a national sample, the main causes of collapsing glomerulopathy are related to infectious diseases, and it affects young patients, especially black and brown men.
- The progression to renal replacement therapy was on average three months after the onset of symptoms.
- We addressed an important gap in the national prevalence in Brazil, aetiology, and prognosis of collapsing glomerulopathy, allowing a better understanding of the disease in future studies.

## Graphical abstract

This multicenter study describes the characterization and outcomes of a retrospective cohort of collapsing glomerulopathy cases conducted in Brazil. These findings reinforce the understanding of this rare glomerulopathy.

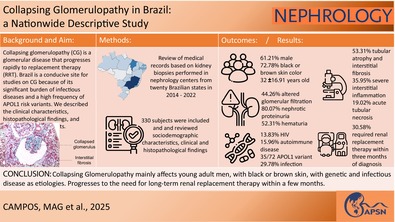

## 1 Introduction

Collapsing glomerulopathy (CG) is a severe glomerular disorder linked to chronic kidney disease (CKD); it occurs in 50%–100% of cases [1, 2]. CG is characterised by segmental collapse of the glomerulus and podocyte hyperplasia [3]. It affects young adults more frequently, with a predilection for African‐American men, despite rare reports of cases among infants and the elderly [4, 5]. The first description of this condition in the 1980s considered it a variant of focal segmental glomerulosclerosis (FSGS) [6]; however, the identification of different molecular and cellular pathways related to disease expression and a greater chance of developing permanent and progressive kidney lesions supported the idea that it is in fact a distinct variant [7]. As CG is a rare condition, there are data gaps regarding geographic distribution, clinical results, and population‐based prognostic studies, despite early advances in therapy [3].

CG has a global distribution, but its primary presentation is most frequent in the African, African‐American, and Caribbean ethnic groups [7]. Although less prevalent in Europeans, CG can develop secondary to other conditions [8]. Many different primary/idiopathic, genetic, and reactive/secondary etiologies have been reported for CG [7]. The classic association with the histological morphology of human immunodeficiency virus (HIV)‐related nephropathy leads to nephrotic syndrome and rapid loss of renal function [9]. The identification of high‐risk variants of *APOL1* alleles increases the risk of developing CG in patients with or without HIV infection [10]. Various other conditions, such as systemic lupus erythematosus (SLE) and infections, are related to CG, including arboviroses, pulmonary tuberculosis, leishmaniasis, parvovirus, cytomegalovirus, Epstein–Barr virus, and, more recently, SARS‐Cov‐2 [11].

In recent years, the incidence of CG associated with infections, chemical agents, and other environmental factors has increased [12]. Thus, the Brazilian population shows considerable potential for the study of CG, owing to the high incidence of infectious diseases [13] and risk populations with an elevated prevalence of the *APOL1* high‐risk genotype [14].

This Brazilian multicentre study aimed to describe sociodemographic, clinical, and histopathological data from the largest population sample reported in the literature.

## 2 Methods

This multicentre retrospective cohort study was conducted between January 2014 and July 2022 and included individuals diagnosed with CG based on native kidney biopsy (KB). The study was conducted in 18 hospitals distributed across all Brazilian regions and included patients from 20 different states and the Federal District.

The inclusion criterion for a participating centre was to be part of the Collapsing Glomerulopathy Brazilian Consortium, in which university hospitals of the Brazilian Company of Hospital Services and other private/public hospitals for nephrology referrals in the country. All centres were responsible for performing KBs and registering medical records. Histopathological analysis of the KB material was performed at the Nephropathology Institute of Minas Gerais, Clínicas Hospital of the Botucatu Medical School, Clínicas Hospital of the University of Sao Paulo Medical School, Clinical Hospital of the Medicine College of Federal University of Pernambuco, Ana Nery Hospital, and University Hospital of Federal University of Maranhão. Other KB collection centres were Vale do São Francisco University Hospital, Professor Alberto Antunes Hospital, Brasília University Hospital, Maria Aparecida Predossian Hospital, Lauro Wanderley Hospital, Alcides Carneiro Hospital, University Hospital of Federal University of Piauí, Clementino Fraga Filho Hospital, Onofre Lopes Hospital, Cariri Regional Hospital, University Hospital of Federal University of Sergipe, and Carlos Macieira Hospital. All the centres care for patients in the public health system and private care (e.g., the Nephropathology Institute, MG) and are the main sites for adult and paediatric nephrology care in their respective regions, from cities to neighbouring states.

KB descriptions were collected from the medical records. KB samples were examined under an optical microscope using haematoxylin–eosin (HE), periodic acid‐Schiff (PAS), Masson's trichrome, and Jones' methenamine silver (JMS) stains. CG diagnosis requires at least one glomerulus presenting with segmental or global collapse with hypertrophy and/or hyperplasia of podocytes (according to the Columbia classification) [6]. In the routine of the participating centres, there is no established protocol for performing electron microscopy (EM), or the technique was not available. Therefore, EM was not performed to define the diagnosis.

Patient identification, data regarding clinical presentation, histopathological characterisation, and follow‐up were collected at least 1 year after diagnosis. Age stratification focused on individuals aged < 60 years (most of the population). Initial values for serum creatinine and 24‐h proteinuria were recorded, such as the time between symptom onset and KB. The estimated glomerular filtration rate (eGFR) was estimated according to the CKD‐EPI equation 2021 [15]. Loss of renal function was considered at the time of KB when the patient showed an eGFR alteration meeting the criteria for acute kidney injury according to the Kidney Disease Improving Global Outcomes (KDIGO) [16] or when the patient presented with serum creatinine > 2.0 mg/dL. Nephrotic proteinuria was defined as the urine protein level than 3.5 g per 24 h [17]. Investigation of the secondary aetiology was performed in some cases, but there was no standardisation of the investigation process among the centres. APOL1 genotyping was performed in 72 patients from 2 centres.

Other histopathological features analysed were the total glomeruli count, number of glomeruli with collapsing lesions (partial or total), and number of globally sclerosed glomeruli. Interstitial fibrosis and inflammation, acute tubular necrosis (ATN), and vascular damage were also evaluated semi‐quantitatively according to the Banff criteria [18] as absent, mild, moderate, or severe. Samples had also been collected for immunofluorescence study with antibodies anti‐IgM, C3, IgA, IgG, and C1q graduated according to the intensity, from “0” for absent to “+3” for brightly reactive.

The monthly clinical follow‐ups were reported. Medications used were divided into classes. After 1 year, creatinine levels and 24‐h proteinuria were measured, as well as the possibility of evolution to permanent renal replacement therapy (RRT) and time of RRT initiation. A Kaplan–Meier curve was performed to demonstrate progression to RRT at 24 months for those patients who had more detailed follow‐up information.

The data were organised using a secure web platform used by hospitals participating in the Collapsing Glomerulopathy Brazilian Consortium. Continuous variables were expressed as median and interquartile range (IQR) or mean and standard deviation (SD), according to the normal distribution of values. Categorical variables were presented as frequencies. We ignored missing data when demonstrating frequencies and proportions in descriptive analysis. The number of data points included for each variable is shown in the results tables. The data were analysed using R version 4.2.2. For the spatial distribution, QGIS version 3.12.0 was utilised. This study was approved by the Institutional Review Board of HUUFMA (statement 4.750.825) and the Faculty of Health and Human Ecology of Minas Gerais (statement 4.153.427).

## 3 Results

**FIGURE 1:**
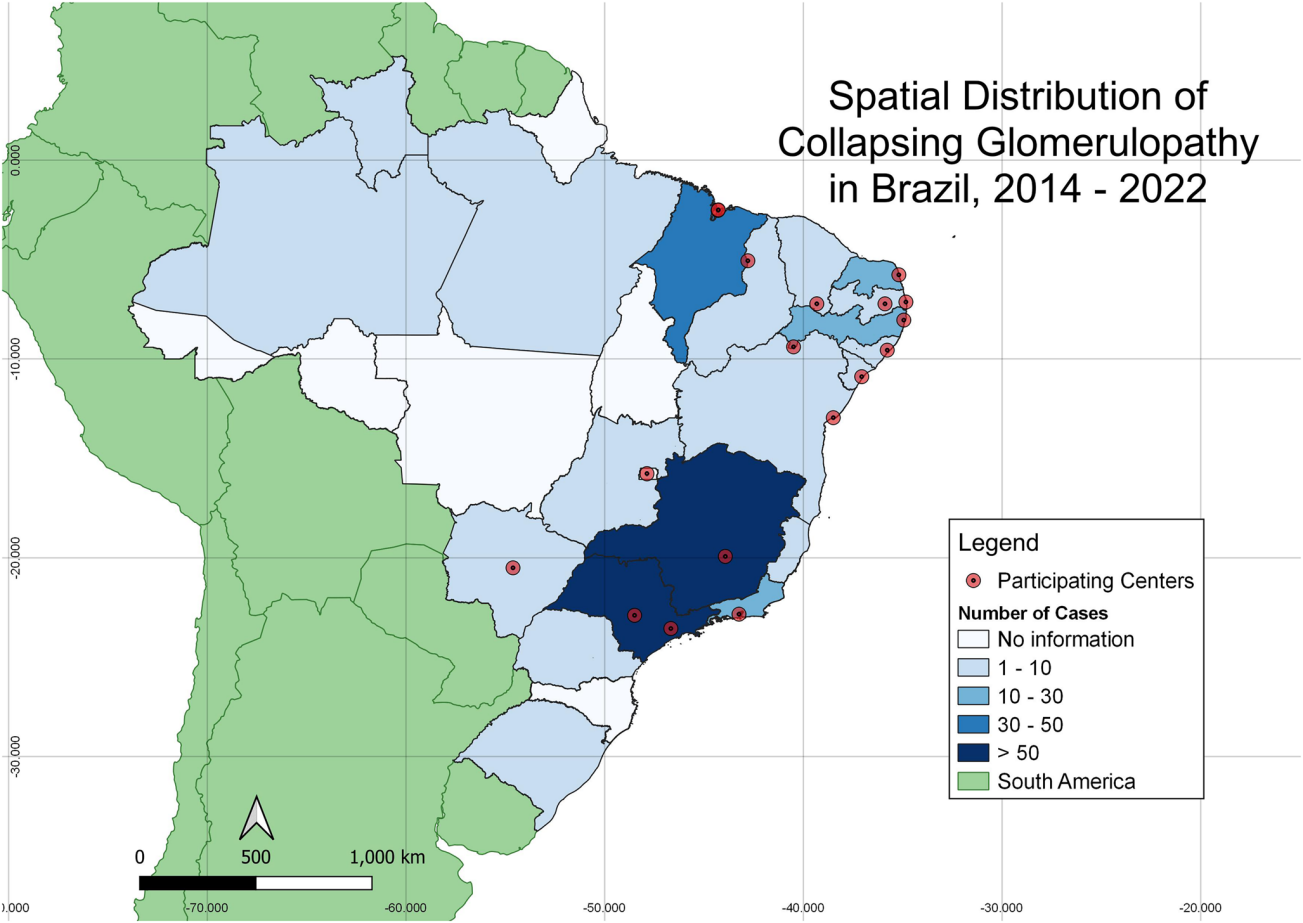
Spatial distribution of Collapsing Glomerulopathy cases in Brazil, between 2014 and 2022. Location of participating centres where data was collected are highlighted in red.

A total of 330 KBs of patients diagnosed with CG were included in the study. Patient data were obtained from 20 of the 26 Brazilian states and the Federal District. Figure 1 shows the spatial distribution and absolute number of cases during the study period and highlights the location of each participating centre. Most patients were from the Southeast region (122, 36.97%) (Table 1).

**TABLE 1:** Sociodemographic characterisation.

Variable	*N* = 330
Region	
Southeast	178 (53.94%)
Northeast	122 (36.97%)
Centre‐west	21 (6.48%)
North	5 (1.52%)
South	4 (1.21%)
Ethnic group	
Black, brown	131 (72.78%)
White	49 (27.22%)
Sex	
Male	202 (61.21%)
Female	128 (38.79%)
Mean age in years (SD)	32 (16.91)
Age group	
< 5 years	7 (2.59%)
5–15 years	28 (10.37%)
16–30 years	116 (42.96%)
31–60 years	98 (36.30%)
> 60 years	21 (7.78%)
Mean time in months (SD) from symptoms onset to kidney biopsy	4 (4.73)
Median initial creatinine level in mg/dl (IQR)	1.74 (1.13–3.17)
Median initial proteinuria in g/24 h (IQR)	7.66 (4.37–12.70)
Presence of hematuria	113 (52.31%)
**Aetiology**	***N* = 94**
HIV	13 (13.83%)
SLE	13 (13.83%)
*APOL1* high‐risk genotype (*N* = 72)	35 (48.61%)
Pregnancy/Postpartum	10 (10.06%)
Lymphoproliferative diseases	8 (8.51%)
Dengue	7 (7.45%)
Viral hepatitis (B and C)	6 (6.39%)
NSAID	5 (5.32%)
Other genetic causes	5 (5.32%)
Systemic Arterial Hypertension	4 (4.25%)
Chikungunya	4 (4.25%)
COVID‐19	4 (4.25%)
Other infections	4 (4.25%)
Other drugs	4 (4.25%)
Diabetes mellitus type 2	3 (3.19%)
Obesity	3 (3.19%)
Cocaine	3 (3.19%)
IgA Nephropathy	3 (3.19%)
Parvovirus B19	3 (3.19%)
Anabolic steroids	2 (2.13%)
Other autoimmune diseases	2 (2.13%)
Neoplasms	2 (2.13%)

*Note:* Laboratory and etiologic data of patients with Collapsing Glomerulopathy in Brazil between 2010 and 2022. Ignored data was not demonstrated.

Abbreviations: HIV: Human immunodeficiency virus; IQR: Interquartile range; NSAID: Non‐steroidal anti‐inflammatory drugs; SD: standard deviation; SLE: Systemic Lupus Erytematosum.

The majority of participants were male (202/330, 61.16%). Each individual's sociodemographic information was not available in the medical records. Non‐white was the most common ethnicity (131/180, 72.78%). A majority of the patients with CG were young adults (mean 32 ± 16.91 years), with 116/270 (42.96%) patients aged 16–30 years.

The mean time between symptom onset and KB was 4 ± 4.73 months (77/330 had this information). At the initial symptoms, the patients already showed an altered eGFR in 104/235 (44.26%) patients and nephrotic proteinuria in 237/296 (80.07%) (Table 2).

**TABLE 2:** Histopathological findings.

Variable	*N* = 330
Interstitial fibrosis	
Absent	18 (5.96%)
Mild	161 (53.31%)
Moderate	67 (22.19%)
Severe	56 (18.54%)
Inflammation scale	
Absent	24 (7.84%)
Mild	172 (56.21%)
Moderate	62 (20.26%)
Severe	48 (15.69%)
Vascular damage	164 (53.77%)
Acute tubular necrosis	58 (19.02%)
IgM	
Absent	60 (24.49%)
1+	95 (38.78%)
2+	62 (25.31%)
3+	28 (11.43%)
C3	
Absent	44 (18.72%)
1+	59 (25.11%)
2+	75 (31.91%)
3+	57 (24.26%)
IgA	
Absent	135 (81.82%)
1+	16 (9.70%)
2+	10 (6.06%)
3+	4 (2.42%)
IgG	
Absent	138 (81.82%)
1+	19 (10.73%)
2+	13 (7.34%)
3+	7 (3.95%)
C1q	
Absent	98 (57.65%)
1+	44 (25.88%)
2+	23 (13.53%)
3+	23 (13.53%)
Mean number of total glomeruli (SD) sampled by biopsy	19 (12.68)
Mean number of globally sclerotic glomeruli (SD) by biopsy	4 (7.88)
Mean number of total viable glomeruli (SD) by biopsy	12 (8.46)
Mean number of affected glomeruli (SD) by biopsy	6 (5.60)

*Note:* Histopathological characterisation of kidney biopsies with Collapsing Glomerulopathy in Brazil between 2014 and 2022. Ignored data was not demonstrated.

Abbreviation: SD: standard deviation.

The aetiology investigation was completed in some patients (94/330). Thus, 28.48% had secondary CG, and 71.52% had primary CG. Of the patients with an identifiable aetiology, 18 patients had more than one potential cause for developing CG. Serology for HIV was positive in 13.83% of the patients, and serology for dengue virus was positive in 7.45% of patients. Other infections such as arbovirosis, COVID‐19, and viral hepatitis were found to be related to the aetiology of GC (Table 2). The APOL1 high‐risk variant was identified in 48.61% (35/72) of patients tested. SLE (13.83%) was the most frequent autoimmune cause. Lymphoproliferative disease (8.51%), mostly lymphoma and multiple myeloma, was described. Pregnancy and postpartum were reported in 10.06% of the cases (Table 2).

Among the 35 patients positive for high‐risk APOL1 alleles, 51.4% were female, and the mean age was 23.94 ± 9.54 years. Median serum creatinine was 1.32 mg/dL (IQR 0.87–1.98), CKD‐EPI eGFR 62.43 mL/min/1.73 m^2^ (IQR 41.15–103.70), and proteinuria 8.00 g/day (IQR 4.63–12.00). Of this sample, 14 (40%) required RRT in the first 36 months.

**FIGURE 2:**
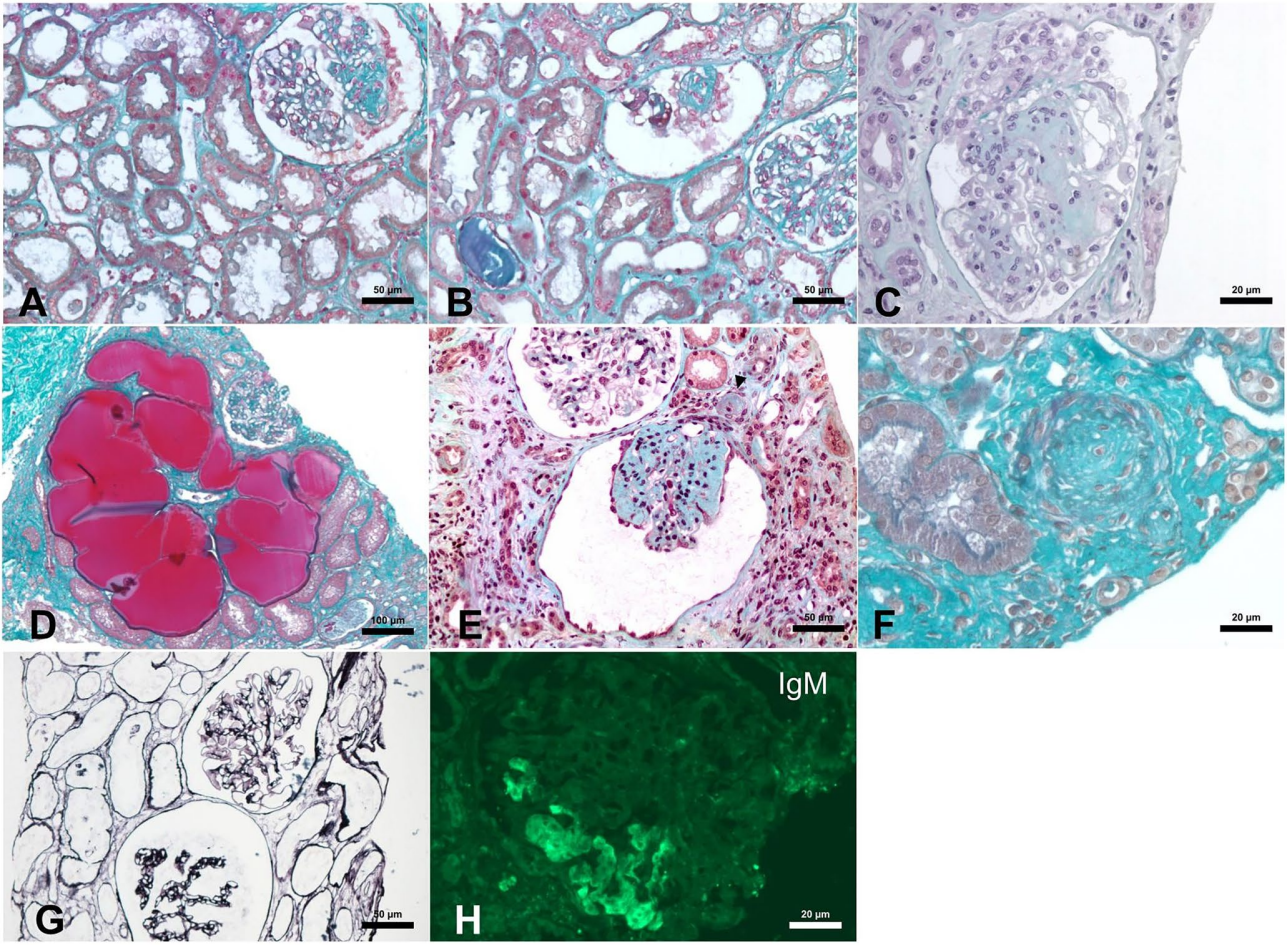
Light microscopy, Masson's trichrome staining (A–F), Jones' methenamine silver staining (G), immunofluorescence (H). (A) Glomerulus with sclerosis of the mesangial matrix and segmental collapse. There is hypertrophy and hyperplasia of podocytes with vacuolated cytoplasm. (B) A typical collapsed glomerulus sided by a normal one, reflecting the focal nature of the disease. (C) Near global sclerosis of a glomerulus. Along with the hypertrophy and hyperplasia of podocytes, the detachment of cells into Bowman space is another typical feature of Collapsing Glomerulopathy. (D) Enlarged tubules with moulding of the contours and a glomerulus with marked podocyte hyperplasia. (E) Adjacent to a totally collapsed glomerulus there is a normal glomerulus. The cortex shows interstitial fibrosis and an arteriole (arrowhead) occluded by hyalinosis. (F) Thrombotic microangiopathy in an artery occluded by concentric hyperplasia. (G) The Jones' methenamine silver stain highlights the collapse of the mesangial apparatus. (H) The immunofluorescence study shows entrapment of IgM in the sclerotic areas.

Regarding histopathological findings, interstitial fibrosis occurred in 94.04% of cases. Interstitial inflammation was graded as moderate/severe in 35.95% of the KBs. Vascular damage was reported in 53.85%. ATN was observed in 53.77% of patients. Immunofluorescence analysis revealed strong positivity for immunoglobulin M (IgM), C3, and C1q. (Table 2). Representative histopathological images of the samples are shown in Figure 2.

**FIGURE 3:**
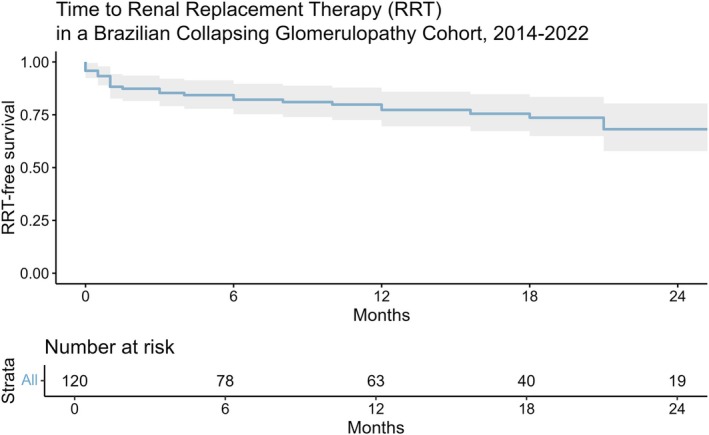
A Kaplan–Meier curve for time to renal replacement therapy among collapsing glomerulopathy patients in a multicenter retrospective cohort study, 2014–2022.

Most of the patients received corticosteroids (74.40%). The creatinine and proteinuria levels remained high even after the follow‐up period of 13 months, IQR: 5.00–21.25 (Table 3). Two patients died during follow‐up owing to complications of acute kidney injury. Of the 121 patients for whom information about the necessity of long‐term RRT was available, 37 (30.58%) ended up using some method of RRT, almost all of whom were undergoing haemodialysis. The median time for this outcome was 3 months (IQR: 1–8) (Table 3). A Kaplan–Meier curve for time to RRT is shown in Figure 3.

**TABLE 3:** Treatment and clinical outcomes.

Variable	*N* = 330
Corticosteroids	93 (74.40%)
Calcineurin inhibitors	45 (41.67%)
Median creatinine (mg/dl) at the end of follow‐up (IQR)	1.83 (1.16–3.03)
Median proteinuria (g/24 h) at the end of follow‐up (IQR)	1.75 (0.4–3.95)
Evolution to RRT	37 (30.58%)
Median time (months) from diagnosis to RRT (IQR)	3.00 (1.00–8.00)
Median time (months) of follow‐up (IQR)	13.00 (5.00–21.25)

*Note:* Clinical outcomes of patients with Collapsing Glomerulopathy in Brazil, between 2014 and 2022. Ignored data was not demonstrated.

Abbreviations: IQR: Interquartil range; RRT: renal replacement therapy.

## 4 Discussion

This nationwide study included a large number of CG patients in Brazil. This suggests that CG affects young patients, mostly adult men with black or brown skin, diagnosed in the advanced stages of the disease, with a rapid evolution to RRT.

The prevalence of CG varies widely among countries and can be difficult to measure because CG and FSGS are often analysed together [19]. Usually, published studies on CG include fewer than 50 cases [20].

In Brazil, the Registry of Glomerulopathy of the Clínicas Hospital of the University of São Paulo Medical School indicates that 36.6% of FSGS cases (48/131) represent patients with CG [21]. In another survey conducted in the Northeast region of Brazil, a region with a lower income, CG represented 3% [12] of primary glomerulopathies in a database of 1151 KBs [18].

In some regions such as Portugal, a study of 6130 KBs carried out since 1981 found only 18 cases of CG, but showed a relationship with HIV and other infections affecting young African‐American patients [22]. A study conducted between 1979 and 1993 in Chicago suggested a gradual increase in the prevalence of CG over the decades [23].

Within the diverse spectrum of HIV‐related renal impairments, CG is classically associated with a rapidly progressive form of eGFR loss in patients with HIV [24]. Although the renal survival of HIV and non‐HIV patients with CG is not significantly different, the predicted risk factors for poor renal survival in CG are severe interstitial fibrosis > 20%, elevated serum creatinine > 2.0 mg/dL, proteinuria > 8 g/24 h, and a low percentage of collapsed glomeruli < 20% [25].

Among non‐HIV patients, CG is associated with autoimmune conditions, lymphoproliferative disease, malignancy, medications, and viral infections [20, 25]. In some cases, CG, a renal manifestation of autoimmune disorders, can precede the appearance of multisystem inflammatory symptoms by a few years [26]. In this study, SLE was the most common autoimmune disease. The relationship between CG and SLE remains unknown and may indicate an extreme form of lupus podocytopathy with rapid progression to end‐stage kidney disease (ESKD) [27].

Nonclassical associations with CG were also observed in our sample. In the case of anabolic steroids, the identification of CG in athletes with rapid progression of renal disease requiring RRT after intake of doping substances has been described [28]. We also demonstrated that CG causes anasarca, high creatinine levels, hypoalbuminemia, and albuminuria in a postpartum woman who was successfully treated with tacrolimus [20]. In this context, the KB was essential for a precise diagnosis [20].

Apart from HIV, other common viral infections associated with CG include Epstein–Barr virus, parvovirus B19, coxsackievirus, cytomegalovirus, human T‐cell lymphotropic virus type 1, hepatitis C virus, and SARS‐CoV‐2 [29, 30, 31, 32, 33]. We also observed a relationship with dengue and chikungunya infections.

Recent outbreaks of new infectious diseases, as well as of COVID‐19, in which the kidney is a targeted organ, will probably increase the number of CG cases in the next few years [34]. As observed in our study, infectious causes were the most common aetiology. Arboviroses are very common in Brazil and are well‐established causes of CG, with some case series of dengue and chikungunya reported [35, 36]. It is believed that the expression of certain viral genes inside kidney cells can lead to the proliferation and differentiation of podocytes, with apoptosis and fibrosis [37]. Evidence is available of a direct viral effect on basic cellular functions in podocytes caused by the systemic inflammatory cascade activated during acute viral infections [38].

Kidney damage related to COVID‐19 disease is well established in the literature [39]. Whether in native or allograft kidneys, CG is the most frequent glomerular disease likely related to COVID‐19 [40]. The proposed term “COVID‐19 associated nephropathy” or COVAN is also influenced by APOL1 high‐risk genotypes as a risk factor for severity [40, 41]. The prognosis of COVAN is poor, with most patients developing advanced CKD and one‐third of patients developing ESKD or death in less than a year [42]. Supportive treatment and high‐dose prednisone appear to have beneficial effects in preventing disease progression [43].

In addition to viral infections, CG has been associated with a form of extrapulmonary tuberculosis in the lymph node [44], filariasis [45], leishmaniasis [46], and *Campylobacter enteritis* [47].

Factors associated with African ancestry in Brazil may explain the higher prevalence of APOL1 risk alleles in our population and, therefore, the higher incidence of severe glomerulopathies affecting young patients, such as CG [48, 49]. This may also explain the greater severity of kidney damage in this population when associated with other diseases, such as COVID‐19 [50].

In a Brazilian cohort of patients with idiopathic CG, APOL1 high‐risk genotypes were detected in 33/70 patients and were associated with a higher proportion of African genetic ancestry in patients aged 9–44 years [51]. CG has also been associated with Mendelian variants (8/70) that can lead to worse kidney prognosis, and genetic status is an independent risk factor for progression to ESKD by 36 months [51].

Despite its most common association with young Afro‐American patients with APOL1 high‐risk genotype, CG can affect elderly Caucasian patients, as it can be associated with conditions such as IgA nephritis or Henoch‐Schönlein purpura [52].

The interaction between infectious etiologies and APOL1 risk alleles in CG is centrally described by the “two‐hit” phenomenon [53]. CG can result from complex interactions between host genetics and environmental triggers, which often include viral infections [3]. The genetic predisposition is conferred by APOL1 high‐risk variants (G1 and G2), which are highly prevalent in populations of African descent [51]. These risk variants constitute the first hit, making podocytes highly susceptible to subsequent injury. Various viral infections serve as the necessary “second hit” (or trigger), including the well‐established associations with HIV (leading to HIV‐associated nephropathy), Parvovirus B19, and most recently, SARS‐CoV‐2 [53].

The proposed pathogenic mechanism for this interaction suggests that the infectious trigger activates a damaging host response. In the context of SARS‐CoV‐2, kidney biopsies from CG patients with high‐risk APOL1 genotypes showed no evidence of direct viral infection (SARS‐CoV‐2 RNA or viral particles were absent) [53].

Instead, the biopsies demonstrated elevated chemokine gene expression and other gene changes consistent with an immune reaction. This supports the hypothesis that the environmental hit initiates a cytokine‐mediated systemic response [54].

Specifically, the genetic susceptibility combined with the increased endogenous production of Th1 cytokines, particularly interferon, in response to the virus, may stimulate the production of a cell‐toxic APOL1 variant protein [55, 56, 57]. This toxicity leads to podocyte injury and the development of CG, underscoring that individuals with the APOL1 high‐risk genotype are at increased risk for an aggressive form of kidney disease when infected with specific viruses [3].

As demonstrated in our study, CG presents with severe proteinuria and a poor prognosis in most cases [58]. One‐third of the patients underwent RRT, and most continued to have high levels of proteinuria and a low creatinine clearance rate during the follow‐up. As observed on KB, interstitial fibrosis was often severe, and globally sclerotic glomeruli were found. Various theories have been proposed to explain the mechanisms of segmental or global glomerular collapse of the basement membranes of glomerular capillaries and podocytes. The loss of actin from the cytoskeleton and a dedifferentiated phenotype can occur. This leads to the disappearance of mature podocyte markers and the development of a proliferative phenotype. Other hypotheses focus on mitochondrial disturbances that explain glomerular collapse, as it has been suggested that podocytes are prone to damage under hypoxia [8].

Patients with CG have worse renal function survival than those with FSGS [59]. Most patients with CG present refractory proteinuria, severe loss of kidney function, and progression to permanent RRT [60]. While other subtypes of FSGS have a kidney survival time of 62.5 months, CG has a kidney survival time of 13 months [1]. However, in the present study, the mean time to progression to RRT was 3 months.

The prognostic of the CG depends on the etiological condition associated [12, 61], with an incidence of end‐stage renal disease ranging from 50%–100% [7, 31, 62, 63]. This poor outcome can be predicted by serum creatinine concentration at the time of biopsy and lack of remission of proteinuria [1]. Furthermore, the risk for renal failure was increased when correlated with the severity of tubular degenerative and regenerative changes, male sex [12], serum creatinine level, proteinuria, interstitial fibrosis, glomeruli with collapsing lesions > 20%, and HIV infection [25].

This retrospective study presented limitations regarding the lack of standardisation of etiologic investigation across participating centres, which makes it impossible to conclude whether it is related to a secondary disorder, if it is an idiopathic condition, or, for example, untested patients for APOL1 genotyping due to limited access to the test. Furthermore, the data were collected from various centres scattered throughout Brazil; therefore, the lack of a unified medical records system and the absence of information, especially prognostic information, were factors. Starting with this study, it will be possible to expand new investigations into the associated factors and long‐term outcomes in patients with CG.

## 5 Conclusion

CG is an aggressive disease with unique characteristics that allow us to consider it as an entity distinct from FSGS. Most patients were young men who progressed rapidly to RRT. In this multicenter nationwide study with longitudinal data, characterisation of clinical and histopathological findings in patients diagnosed with CG was possible, making it the largest population CG study reported to date.

## Funding

The authors have nothing to report.

## Disclosure

The authors have nothing to report.

## Conflicts of Interest

The authors declare no conflicts of interest.

## Data Availability

The database is publicly available and published in an online repository; see https://doi.org/10.6084/m9.figshare.27229713.v2.
